# Supplementary material for: Accuracy and real time optimization of remote sensing image change detection based on IRAU and DSC
Source: PLoS One. 2025 Aug 13;20(8):e0329447. doi: 10.1371/journal.pone.0329447 (PMC12349244; doi:10.1371/journal.pone.0329447)
Supplement: S1 File — (DOC) [file pone.0329447.s001.doc]

**The data in Figure 9** (a)

|  | M1 | M2 | M3 | M4 | IRAU-DSC |
| --- | --- | --- | --- | --- | --- |
| 0 | 0.47 | 0.25 | 0.43 | 0.32 | 0.48 |
| 100 | 0.47 | 0.19 | 0.44 | 0.33 | 0.56 |
| 200 | 0.4 | 0.21 | 0.41 | 0.32 | 0.55 |
| 300 | 0.39 | 0.26 | 0.37 | 0.35 | 0.49 |
| 400 | 0.47 | 0.2 | 0.4 | 0.32 | 0.51 |
| 500 | 0.38 | 0.15 | 0.4 | 0.32 | 0.59 |
| 600 | 0.44 | 0.22 | 0.39 | 0.35 | 0.46 |
| 700 | 0.42 | 0.26 | 0.41 | 0.26 | 0.46 |
| 800 | 0.42 | 0.3 | 0.39 | 0.28 | 0.49 |
| 900 | 0.41 | 0.26 | 0.4 | 0.26 | 0.47 |
| 1000 | 0.45 | 0.2 | 0.4 | 0.34 | 0.51 |
| 1100 | 0.45 | 0.18 | 0.42 | 0.34 | 0.49 |
| 1200 | 0.42 | 0.23 | 0.37 | 0.29 | 0.49 |
| 1300 | 0.44 | 0.25 | 0.43 | 0.32 | 0.49 |
| 1400 | 0.41 | 0.2 | 0.39 | 0.26 | 0.51 |
| 1500 | 0.41 | 0.23 | 0.39 | 0.32 | 0.48 |
| 1600 | 0.46 | 0.3 | 0.38 | 0.25 | 0.51 |
| 1700 | 0.61 | 0.15 | 0.36 | 0.25 | 0.73 |
| 1800 | 0.62 | 0.21 | 0.38 | 0.25 | 0.775 |
| 1900 | 0.63 | 0.28 | 0.42 | 0.34 | 0.82 |
| 2000 | 0.68 | 0.21 | 0.48 | 0.31 | 0.95 |
| 2100 | 0.71 | 0.17 | 0.53 | 0.32 | 0.97 |
| 2200 | 0.94 | 0.24 | 0.73 | 0.27 | 0.95 |
| 2300 | 0.95 | 0.17 | 0.75 | 0.34 | 0.98 |
| 2400 | 0.95 | 0.19 | 0.91 | 0.5 | 0.98 |
| 2500 | 0.91 | 0.29 | 0.91 | 0.56 | 0.98 |
| 2600 | 0.95 | 0.15 | 0.9 | 0.57 | 0.97 |
| 2700 | 0.94 | 0.18 | 0.94 | 0.59 | 0.97 |
| 2800 | 0.9 | 0.28 | 0.92 | 0.6 | 0.99 |
| 2900 | 0.91 | 0.3 | 0.94 | 0.75 | 0.95 |
| 3000 | 0.93 | 0.27 | 0.92 | 0.79 | 0.96 |
| 3100 | 0.95 | 0.21 | 0.87 | 0.85 | 0.97 |
| 3200 | 0.95 | 0.35 | 0.93 | 0.79 | 0.96 |
| 3300 | 0.96 | 0.36 | 0.89 | 0.82 | 0.95 |
| 3400 | 0.94 | 0.36 | 0.87 | 0.86 | 0.96 |
| 3500 | 0.91 | 0.41 | 0.89 | 0.8 | 0.95 |
| 3600 | 0.92 | 0.44 | 0.92 | 0.84 | 0.98 |
| 3700 | 0.91 | 0.71 | 0.91 | 0.83 | 0.98 |
| 3800 | 0.95 | 0.67 | 0.89 | 0.86 | 0.97 |
| 3900 | 0.92 | 0.66 | 0.94 | 0.84 | 0.97 |

**The data in Figure 9 (b**)

|  | before | after |
| --- | --- | --- |
| IRAU-DSC | 0.54 | 0.97 |
| M4 | 0.36 | 0.83 |
| M3 | 0.44 | 0.91 |
| M2 | 0.25 | 0.68 |
| M1 | 0.48 | 0.93 |

**The data in Figure 10**

|  | IRAU-DSC | M4 | M3 | M2 | M1 |
| --- | --- | --- | --- | --- | --- |
| 1 | 95.9 | 86.08 | 91.13 | 70.34 | 93.25 |
| 2 | 98.14 | 87.01 | 90.33 | 68.55 | 94.78 |
| 3 | 97.39 | 85.13 | 90.73 | 67.53 | 94.61 |
| 4 | 96.04 | 85.3 | 91.08 | 71.71 | 95.91 |
| 5 | 99.04 | 83.12 | 91.47 | 71.97 | 93.91 |
| 6 | 97.55 | 88.37 | 92.18 | 70.84 | 94.24 |
| 7 | 99.33 | 88.17 | 90.27 | 71.19 | 94.91 |
| 8 | 97.41 | 83.47 | 90.04 | 68.34 | 93.62 |
| 9 | 97.46 | 87.21 | 90.93 | 76.85 | 95.2 |
| 10 | 96.29 | 85.19 | 90 | 74.76 | 93.33 |
| 11 | 96.26 | 84.1 | 90.66 | 69.46 | 94.82 |
| 12 | 96.01 | 88.93 | 91.14 | 72.44 | 95.54 |
| 13 | 97.4 | 85.34 | 90.12 | 68.46 | 94.7 |
| 14 | 95.82 | 87.44 | 91.26 | 71.22 | 95.93 |
| 15 | 97.07 | 84.44 | 90.59 | 76.1 | 95.89 |
| 16 | 98.67 | 85.05 | 93.17 | 69.58 | 94.83 |
| 17 | 98.42 | 87.74 | 90.38 | 76.94 | 94.48 |
| 18 | 97.86 | 88.61 | 90.57 | 70.62 | 95.81 |
| 19 | 99.68 | 84.23 | 90.12 | 76.15 | 94.08 |
| 20 | 99.62 | 83.25 | 92.04 | 76.27 | 93.72 |
| Means | 94.68 | 71.97 | 90.91 | 85.91 | 97.57 |

**The data in Figure 11**

|  | 0 | 0.1 | 0.2 | 0.3 | 0.4 | 0.5 | 0.6 | 0.7 | 0.8 | 0.9 | 1 |
| --- | --- | --- | --- | --- | --- | --- | --- | --- | --- | --- | --- |
| M1 | 0 | 0.43 | 0.75 | 0.88 | 0.95 | 0.99 | 1 | 1 | 1 | 1 | 1 |
| M2 | 0 | 0.31 | 0.45 | 0.6 | 0.7 | 0.77 | 0.83 | 0.9 | 0.95 | 1 | 1 |
| M3 | 0 | 0.4 | 0.62 | 0.85 | 0.9 | 0.95 | 1 | 1 | 1 | 1 | 1 |
| chance leve | 0 | 0.1 | 0.2 | 0.3 | 0.4 | 0.5 | 0.6 | 0.7 | 0.8 | 0.9 | 1 |
| IRAU-DSC | 0 | 0.62 | 0.91 | 0.98 | 0.99 | 1 | 1 | 1 | 1 | 1 | 1 |
| M4 | 0 | 0.29 | 0.6 | 0.73 | 0.8 | 0.87 | 0.93 | 1 | 1 | 1 | 1 |
| chance leve | 0 | 0.1 | 0.2 | 0.3 | 0.4 | 0.5 | 0.6 | 0.7 | 0.8 | 0.9 | 1 |

The data in Figure 12

|  |  | House | River | Road |
| --- | --- | --- | --- | --- |
| M1 | House | 0.83 | 0.09 | 0.08 |
|  | River | 0.07 | 0.88 | 0.05 |
|  | Road | 0.03 | 0.11 | 0.86 |
| M2 | House | 0.79 | 0.10 | 0.11 |
|  | River | 0.12 | 0.80 | 0.08 |
|  | Road | 0.09 | 0.15 | 0.76 |
| IRAU-DSC | House | 0.93 | 0.04 | 0.03 |
|  | River | 0.03 | 0.91 | 0.06 |
|  | Road | 0.05 | 0.01 | 0.94 |
