# Supplementary material for: Accuracy and real time optimization of remote sensing image change detection based on IRAU and DSC
Source: PLoS One. 2025 Aug 13;20(8):e0329447. doi: 10.1371/journal.pone.0329447 (PMC12349244; doi:10.1371/journal.pone.0329447)
Supplement: Attachment — Submitted filename: Review.doc [file pone.0329447.s002.doc]

Your research idea is promising, but I would suggest elaborating on certain aspects.

1.Since your focus is on remote sensing data, have you considered using different types of remote data (Hyperspectral ,Multispectral) to support and justify your proposed approach.

2.Please include a description of the dataset you used in your study.

3.Briefly state the limitations of your proposed method.
