# Supplementary material for: Accuracy and real time optimization of remote sensing image change detection based on IRAU and DSC
Source: PLoS One. 2025 Aug 13;20(8):e0329447. doi: 10.1371/journal.pone.0329447 (PMC12349244; doi:10.1371/journal.pone.0329447)
Supplement: Attachment — Submitted filename: Response to reviewers comments.docx [file pone.0329447.s003.docx]

Review Comments to the Author

Please use the space provided to explain your answers to the questions above. You may also include additional comments for the author, including concerns about dual publication, research ethics, or publication ethics. (Please upload your review as an attachment if it exceeds 20,000 characters)

Reviewer #1:

1. Please explain about the Data properly. Remote Sensing data must include about the location of the study area, time of data acquisition and also about data source.

Austria Steiermark (Geographical distribution center: longitude 15.0024°, latitude 47.2738°)

Reply: Thank you very much for your suggestion. The details of the dataset including the location of the study area, time of data acquisition and data source have been added. As follows:

The research uses the change detection dataset as a training and test set (8:2) with remotely sensed data from a region in Austria Steiermark (Geographical distribution center: longitude 15.0024°, latitude 47.2738°) covering the period from June 2019 to September 2021, containing Landsat-8 and Sentinel-2 multi-temporal imagery with a resolution of 10-30 meters. The data sources are the U.S. Geological Survey and European Space Agency public databases, covering typical scenarios such as urban sprawl and vegetation changes. The study first trains the model to verify its training efficiency, as presented in Figure 9.

2. Reference citation in methodology is very less. Except your results all the figure captions should be cited with proper References.

Reply: According to your suggestion, all the figure captions have been cited with proper References (except our results), for example:

Figure 1. The structure of IRAU and Res2Net+ block [22]

[22]	F. Chen, N. Wang, B. Yu, and L. Wang, “Res2-Unet: A New Deep Architecture for Building Detection From High Spatial Resolution Images,” IEEE J. Sel. Top. Appl. Earth Obs. Remote Sens., vol. 15, pp. 1494-1501, Jan. 2022, 10.1109/JSTARS.2022.3146430.

. Your Figure 13. must be represented in the form of a map with proper legends highlighting the changes.

Reply: Thanks for your kind suggestion. Figure 13 has been modified to:

**Figure 13. Change detection on a spatial scale**

4. ROC curve will only give the model Accuracy, but what is the Accuracy of your result? (the Change Detection map)

Reply: Thanks for your suggestion. The ROC curve can measure the accuracy of a research model (measuring the model's classification ability). The accuracy of the Change Detection map (the model's prediction results for areas of surface change) is measured by calculating TP, TN, FP, and FN, and then deriving the Kappa coefficient:

And Kappa coefficient can reflect the degree of consistency between the classification results and the real labeling, while excluding the consistency caused by random factors. Therefore, the study compares the Kappa coefficients of different methods to verify their accuracy for change detection maps, and the results are shown in Table 1.

5. You must calculate "Kappa Statistic" or "Kappa Coefficient" for your result accuracy.

Reply: Thanks for your kind suggestion. Kappa Coefficient has been calculated:

**Table 1. Kappa coefficients for different algorithms**

| Methods | TP | TN | FP | FN | Kappa Coefficient |
| --- | --- | --- | --- | --- | --- |
| M1 | 700 | 200 | 50 | 50 | 0.75 |
| M2 | 720 | 180 | 70 | 30 | 0.78 |
| M3 | 680 | 220 | 30 | 70 | 0.72 |
| M4 | 750 | 150 | 100 | 0 | 0.82 |
| IRAU-DSC | 800 | 180 | 20 | 0 | 0.9 |

6. What is the validation of your result with Ground Data?

Reply: Thank you very much for your suggestion. The validation of our result with Ground Data has been explained as follows:

Finally, in order to verify the consistency of the model detection results with the ground data, the study selects the Intersection over Union (IoU), the ratio of correctly categorized pixels (changed/unchanged) to the total pixels (Correct Pixel Ratio), and the proportion of undetected in the real change area (Omission Error), Position Error and Area Error are compared, and the results are shown in Table 5.

Reviewer #2:

Your research idea is promising, but I would suggest elaborating on certain aspects.

1. Since your focus is on remote sensing data, have you considered using different types of remote sensing data(Hyperspectral,multispectral) to support and justify your proposed approach.

Reply: Thanks for your suggestion. Comparison experiments for multispectral scenes have been added:

In addition, in order to verify the generalization and robustness of the research model in different spectral scenarios, the study selects different spectral scenarios for change detection experiments, and the results are shown in Table 4.

**Table 4 Comparison of model performance in different different spectral scenarios**

| Scene conditions | Methods | Accuracy (%) | Time Deviation (ms) | Kappa  Coefficient |
| --- | --- | --- | --- | --- |
| Hyperspectral  (224 bands) | M1 | 86.2 | 34.5 | 0.81 |
|  | M2 | 88.7 | 41.2 | 0.83 |
|  | IRAU-DSC | 93.5 | 28.7 | 0.9 |
| Multi-spectral  (8 bands) | M1 | 82.4 | 28.1 | 0.76 |
|  | M2 | 84.9 | 33.6 | 0.79 |
|  | IRAU-DSC | 89.3 | 24.9 | 0.86 |
| Low resolution multispectral  (4 bands) | M1 | 78.6 | 21.3 | 0.71 |
|  | M2 | 80.2 | 25.8 | 0.73 |
|  | IRAU-DSC | 85.4 | 19.5 | 0.81 |

2. Please include a description of the dataset you used in your study.

Reply: Thank you for your suggestion. The details of the dataset including the location of the study area, time of data acquisition and data source have been added. As follows:

The research uses the change detection dataset as a training and test set (8:2) with remotely sensed data from a region in Western Europe covering the period from June 2019 to September 2021, containing Landsat-8 and Sentinel-2 multi-temporal imagery with a resolution of 10-30 meters. The data sources are the U.S. Geological Survey and European Space Agency public databases, covering typical scenarios such as urban sprawl and vegetation changes. The study first trains the model to verify its training efficiency, as presented in Figure 9.

3. Briefly state the limitations of your proposed method.

Reply: Thanks to your suggestion, the limitations of the study have been added:

However, IRAU relies on multiple modules connected in series with jump connections, which may lead to gradient flow redundancy and reduced feature fusion efficiency. And although DSC reduces the computational volume, the sensory field is limited in high-resolution input, and the fine-grained feature extraction is insufficient. To address the above problems, the research will simplify the module interaction path to optimize the gradient propagation efficiency, design adaptive cavity convolution to enhance the receptive field of DSC, introduce a cross-module dynamic weight allocation mechanism to improve the compatibility of heterogeneous features, and explore the lightweight architecture to balance the computational load and detection accuracy.
